# Tactile Electrosurgical Ablation: A Technique for the Treatment of Intractable Heavy and Prolonged Menstrual Bleeding

**DOI:** 10.1155/2015/895062

**Published:** 2015-07-30

**Authors:** Ali M. El Saman, Faten F. AbdelHafez, Kamal M. Zahran, Hazem Saad, Mohamed Khalaf, Mostafa Hussein, Ibrahim M. A. Hassanin, Saba M. Shugaa Al Deen

**Affiliations:** ^1^Women's Health Center, Department of Obstetrics & Gynecology, Faculty of Medicine, Assiut University, Assiut, Egypt; ^2^Department of Obstetrics & Gynecology, Faculty of Medicine, Sohag University, Sohag, Egypt; ^3^Department of Obstetrics & Gynecology, University Hospital of Sana'a, Yemen

## Abstract

*Objective*. To study the efficacy and safety of tactile electrosurgical ablation (TEA) in stopping a persistent attack of abnormal uterine bleeding not responding to medical and hormonal therapy. *Methods*. This is a case series of 19 cases with intractable abnormal uterine bleeding, who underwent TEA at the Women's Health Center of Assiut University. The outcomes measured were; patient's acceptability, operative time, complications, menstrual outcomes, and reintervention. *Results*. None of the 19 counseled cases refused the TEA procedure which took 6–10 minutes without intraoperative complications. The procedure was successful in the immediate cessation of bleeding in 18 out of 19 cases. During the 24-month follow-up period, 9 cases developed amenorrhea, 5 had scanty menstrual bleeding, 3 were regularly menstruating, 1 case underwent repeat TEA ablation, and one underwent a hysterectomy. *Conclusions*. TEA represents a safe, inexpensive, and successful method for management of uterine bleeding emergencies with additional long-term beneficial effects. However, more studies with more cases and longer follow-up periods are warranted.

## 1. Introduction

Heavy and/or prolonged menstrual bleeding (HMB) stands among the most common presentations in the acute gynecology units and accounts for up to 70% of all gynecological consultations in the peri- and postmenopausal years [1]. Hysterectomy represents the ultimate treatment for HMB; however, it might have its potential psychosexual and depressing effects [2, 3]. In addition, hysterectomy is not an ideal option for medically unfit patients and/or those with deteriorated hemodynamics.

Several reports have proposed first generation endometrial ablation techniques [4–6]; however active bleeding may interfere with the appropriate visualization of the uterine cavity and increase the risk of complications. On the other hand, the second generation ablation devices [7, 8] require less skill but the high cost of their disposables limits their affordability in low income settings.

The authors are working in a setting of limited resources where offering expensive disposables for hysteroscopic ablators is not feasible all the time. They have experience in treating challenging cases of endometrial ablation using a specially designed monopolar electrosurgical coagulation probe able to perform electrosurgical ablation without hysteroscopy [9]. In a series of previous studies, the safety and the feasibility of TEA were investigated using an in vitro model of hysterectomy specimens [9] and a pilot clinical study under laparoscopic monitoring [10]. The experimental results showed complete coagulation of the endometrium along with 2–4 mm of the adjacent myometrium. No full thickness damage was observed, with the maximum depth involving only 16% of myometrial thickness [9]. Laparoscopic monitoring was performed in the initial clinical series to confirm that full thickness damage did not occur [10]. The aim of the present work was to investigate the role of tactile ablation in cases presenting with active uterine bleeding as an emergency minimally invasive procedure under ultrasonographic monitoring.

## 2. Materials and Methods

This study was approved by the ethics committee of Assiut University Hospitals. An institutional review board approval was obtained for using TEA in the management of heavy and/or prolonged menstrual bleeding that failed to respond to medical/hormonal treatment. A thorough history taking and clinical examination were completed for all patients. In addition, a routine ultrasonographic examination, followed by a dilatation and curettage biopsy were performed. Cases who desired further fertility, had a lower segment caesarean scars, with uterine size >10-week pregnancy, and presented with a coexisting gynecological pathology and/or their pathological examination showed atypical hyperplasia were excluded. Eligible cases were counseled regarding the risks, benefits, and available alternatives of the procedure. A written informed consent was then taken.

**Figure 1:**
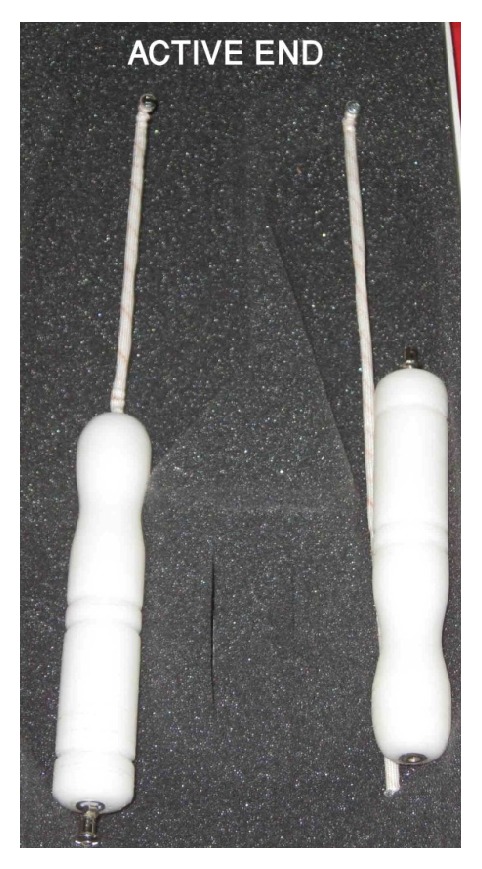
The tactile electrosurgical ablation (TEA) probe.

Under general anesthesia and in lithotomy position, the TEA procedure was preceded by cervical dilatation and uterine curettage to minimize the thickness of the endometrium and enhance the ablation efficacy. The procedure was performed under transabdominal ultrasonographic monitoring. When the uterus was well-visualized in a clear longitudinal scan, the uterine length was measured and the TEA probe was calibrated by sliding the flange depth gauge according to the measured uterine length (Figure 1). The power setting of electrosurgical coagulation unit was adjusted at 60 Watts at the coagulation mode; then the TEA probe was connected to its active monopolar socket.

The active end of TEA probe was introduced through the cervix until it touched the uterine fundus. The TEA probe was then directed to press gently on the anterior wall and the electrosurgical coagulation was activated while the TEA probe was slowly withdrawn down to the internal os. The electrosurgical coagulation was then switched off; the probe was reintroduced until the fundus and the procedure was repeated working from the right to the left side until complete coagulation of the anterior wall. Thereafter, the posterior uterine wall was coagulated in the same manner. Lastly the TEA probe was passed across the fundus slowly working from the right to the left uterine cornual ends to ensure the complete coagulation of the whole endometrium. The US transducer was tilted from side to side to better visualize the TEA probe inside the uterine cavity.

Coagulation was judged complete (first endpoint) when the activated TEA probe was passed over the whole uterine cavity. By the end of uterine cavity coagulation, tissue elasticity was lost and the uterine cavity underwent some shrinkage that impeded the easiness of moving the TEA probe up and down as if the uterine wall was clenching or holding it. This interesting sign was given the name “the grip sign” and it confirmed the fulfillment of the procedure. We theoretically evaluated other endpoints in the form of impending perforation (the occurrence of any penetration of the TEA probe through the myometrium in the ultrasonic scan), suspected or actual perforation, excessive vaginal bleeding, and/or unsatisfactory sonographic monitoring (inability to visualize the uterus in a clear longitudinal scan view with hyperechoic probe inside the uterine cavity between the two uterine walls and its tip below the fundus).

At the end of the procedure and after cessation of uterine bleeding, we performed a diagnostic hysteroscopy, for detection of missed foci of untreated endometrium. All cases were followed up every three months for one year. Longer-term follow-up for another one year was obtained by phone calls.

## 3. Results

Nineteen women presenting with heavy/prolonged menstrual bleeding were included in the current study. Their ages ranged from 40 to 47. The bleeding was heavy and prolonged in 15 cases and was on and off for three months in 2 cases, and continuous mild spotting was observed in the other 2 cases. Preoperative hemoglobin concentration ranged from 7 to 10 gm%. Two cases received preoperative blood transfusion, one received total dose iron infusion, and three cases were on oral iron.

Operative time ranged from 6 to 10 minutes depending on the size of the uterine cavity and proposed end points. No intraoperative difficulties, complications, or full thickness damage was reported. The grip sign was elicited very well in 17 cases and was not so obvious in the remaining 2 cases. Immediate diagnostic hysteroscopy was possible in 15 cases and showed complete coagulation (faint yellowish to dark brown color) apart from diminutive foci of uncoagulated endometrium (pinkish color) near the uterine cornua.

The TEA procedure was successful in the immediate cessation of the bleeding attack in 18 out of the 19 cases. During six- to 24-month follow-up period, 9 cases developed amenorrhea, 5 had light menstrual bleeding, 3 had regular menstruation, one case underwent a repeat ablation 6 months later (on request), and the last one had undergone a hysterectomy for recurrence of HMB.

## 4. Discussion

Tactile electrosurgical ablation (TEA) was successfully performed with satisfactory outcomes for 19 cases with heavy and/or prolonged uterine bleeding during an active, relentless bleeding attack. The TEA procedure was effective in the immediate cessation of the bleeding attack in 18 of the 19 cases. Although hysterectomy remains the definitive treatment for HMB, the associated morbidities are significant especially in medically unfit patients and/or in deteriorated hemodynamic states [9, 10]. The results of an applied patient's questionnaire in a hospital in Netherlands showed that approximately one-third of women undergoing hysterectomy due to abnormal uterine bleeding would have opted for endometrial ablation and 45% would have opted for a levonorgestrel-releasing IUD, despite a risk of 50% possibility of treatment failure [11].

However, for all types of hysteroscopic ablation, satisfactory proper visualization of the uterine cavity is vital for successful and safe performance. In addition, the number of well-trained personnel in hysteroscopic surgeries is still limited. Other challenges and financial constraints in developing countries result in difficulties in maintaining perfectly working hysteroscopic equipment [10].

In the present series, active heavy uterine bleeding was anticipated to result in failure to perform hysteroscopic endometrial ablation safely as it interferes with proper visualization. Other investigators reported such difficulties in hysteroscopic surgery [12]. Thermal balloons and other second generation ablators could play a backup role in such cases. However, financial constraints and scarce health resources limit the availability and affordability of expensive disposables [13, 14].

Hysteroscopic electrosurgeries are performed using appropriate distension media which should be electrolyte-free with monopolar electrosurgical coagulation. Nevertheless, electrolyte-free distension media has its own problems [15–17]. The most vulnerable subjects are those with unclear hysteroscopic view due to excessive bleeding. This requires too much washing at higher pressures, which pushes extra volumes of fluids into the open-mouthed bleeding vessels for a more prolonged duration. The use of TEA has the advantage of avoiding fluid overload especially in this vulnerable group of patients.

The technique of tactile electrosurgical ablation (TEA) is largely similar to the dilatation and curettage procedure. Hence, the procedure requires awareness of electrosurgical principles, satisfactory experience in ultrasonic monitoring, high resolution ultrasonic machine, and adequate experience in performing dilatation and curettage. Even if it seems that TEA is a blind technique, it is not underprivileged of direct external visual monitoring by ultrasonography. In addition, TEA is carried out under the great tactile sense of the experienced gynecologist, promoting the survival of that clinical sense before being a state-of-a-lost art [18].

The main weakness point of the present study is the limited number of cases that makes statistical and power analysis impractical. However, it opens a new perspective for a novel inexpensive backup approach for electrosurgical ablation without distension media, hysteroscopy, or expensive disposables especially in limited resources' settings. Moreover, TEA has the potential to be the procedure of choice in cases with heavy uterine bleeding as it depends on tactile rather than visual ablation and is found to be effective in stopping the active bleeding in 90% of cases.

## 5. Conclusions

Tactile endometrial ablation is a promising inexpensive management procedure of heavy uterine bleeding in low sources' setting. However larger studies are recommended to confirm its safety and cost effectiveness.
